# Staged posterior fossa decompression and microvascular decompression for trigeminal neuralgia in autosomal dominant osteopetrosis type I: A case report

**DOI:** 10.1097/MD.0000000000046754

**Published:** 2025-12-19

**Authors:** Sungsoo Bae, Seung Hoon Lim, Hee Sup Shin, Hak Cheol Ko

**Affiliations:** a Department of Neurosurgery, Kyung Hee University College of Medicine, Kyung Hee University Hospital at Gangdong, Seoul, Republic of Korea.

**Keywords:** microvascular decompression, osteopetrosis, posterior fossa, staged decompression, trigeminal neuralgia

## Abstract

**Rationale:**

Osteopetrosis is a rare hereditary skeletal disorder characterized by increased bone density resulting from defective osteoclast-mediated bone resorption. This condition may lead to cranial nerve compression syndromes, with trigeminal neuralgia (TN) occurring infrequently.

**Patient concerns:**

We report the case of a 26-year-old woman with autosomal dominant osteopetrosis who presented with intractable, electric shock-like facial pain consistent with TN that was refractory to medical therapy.

**Diagnoses:**

Imaging revealed marked posterior fossa crowding with evidence of neurovascular compression of the left trigeminal nerve root entry zone, further compounded by cranial hyperostosis.

**Interventions:**

A staged surgical approach was undertaken. The first stage involved suboccipital craniectomy with C1 laminectomy to decompress the posterior fossa and restore cerebrospinal fluid dynamics. The second stage comprised microvascular decompression via a retrosigmoid approach after optimization of the intracranial space.

**Outcomes:**

Following completion of the staged surgical intervention, the patient achieved complete resolution of facial pain without neurological deficits. Symptom relief was sustained over a 5-year follow-up period, with no evidence of recurrence.

**Lessons:**

A staged decompressive approach represents an effective and durable treatment option for TN secondary to osteopetrosis, particularly in cases with markedly compromised posterior fossa anatomy. Initial posterior fossa decompression facilitates a safer and more effective subsequent microvascular decompression by improving the surgical corridor.

## 1. Introduction

Trigeminal neuralgia (TN) is a severe, paroxysmal facial pain disorder most commonly caused by neurovascular compression at the root entry zone (REZ) of the trigeminal nerve.^[1]^ While medical therapy is generally the first-line treatment, microvascular decompression (MVD) remains the most effective surgical option, particularly in patients with confirmed neurovascular pathology.^[2]^ However, in younger individuals, secondary causes such as tumors, demyelinating disorders, and craniofacial skeletal anomalies, including osteopetrosis, should be carefully considered.^[3,4]^

Autosomal-dominant osteopetrosis (ADO) is a rare hereditary skeletal disorder resulting from defective osteoclast-mediated bone resorption, which leads to excessive bone density and diffuse skeletal sclerosis.^[5]^ Involvement of the skull base may cause foraminal narrowing and cranial nerve compression, producing complications such as optic atrophy, hearing loss, and TN.^[6]^ Because the co-occurrence of TN and ADO is exceedingly uncommon, optimal treatment strategies for these patients remain poorly established.^[7]^

Here, we present a rare case of TN in a patient with ADO type I that was successfully treated using a staged surgical approach. Initial posterior fossa decompression alleviated brainstem crowding and improved cerebrospinal fluid (CSF) dynamics, thereby facilitating a safer and more effective second-stage MVD to relieve neurovascular compression. This case underscores the practical value of a sequential decompression strategy in anatomically restricted secondary TN, expanding the current management paradigm beyond conventional single-stage procedures.

## 2. Case presentation

### 2.1. Patient history and examination

A 26-year-old woman with a known diagnosis of ADO type I presented with a 1-year history of left-sided TN. She experienced paroxysmal, electric shock-like facial pain affecting the maxillary (V2) and mandibular (V3) divisions of the trigeminal nerve. The symptoms were triggered by mild stimuli such as chewing and speaking. Although the pain initially responded to pharmacologic therapy with oxcarbazepine and gabapentin, it gradually became refractory over time. The patient had a significant family history of ADO and exhibited craniofacial features consistent with skeletal dysplasia, including dolichocephaly, mandibular prominence, and midfacial hypoplasia. There were no systemic manifestations such as anemia, recurrent infections, or skeletal fragility, and she exhibited normal stature without a history of fractures. Neurological examination revealed stimulus-triggered facial pain confined to the left V2 and V3 divisions without objective sensory deficits or involvement of other cranial nerves. There were no signs of brainstem compression or cerebellar dysfunction. Fundoscopic examination findings were initially unremarkable; however, subsequent imaging (described below) demonstrated mild hydrocephalus. Collectively, the clinical picture was consistent with TN likely secondary to craniofacial hyperostosis associated with ADO.

### 2.2. Initial imaging prior to first-stage surgery

**Figure 1.**
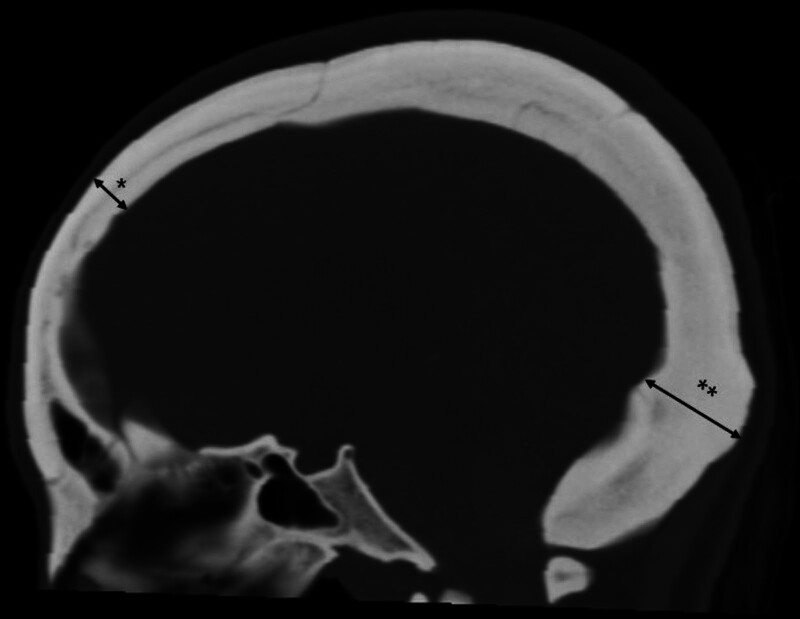
Computed tomography prior to the first-stage decompressive surgery, demonstrating diffuse cranial bone thickening with the characteristic “marble bone” appearance, a diagnostic hallmark of osteopetrosis. Calvarial thickness measures approximately 7 to 8 mm in the frontal region (*) and up to 29 mm in the suboccipital area (**), contributing to significant posterior fossa crowding.

**Figure 2.**
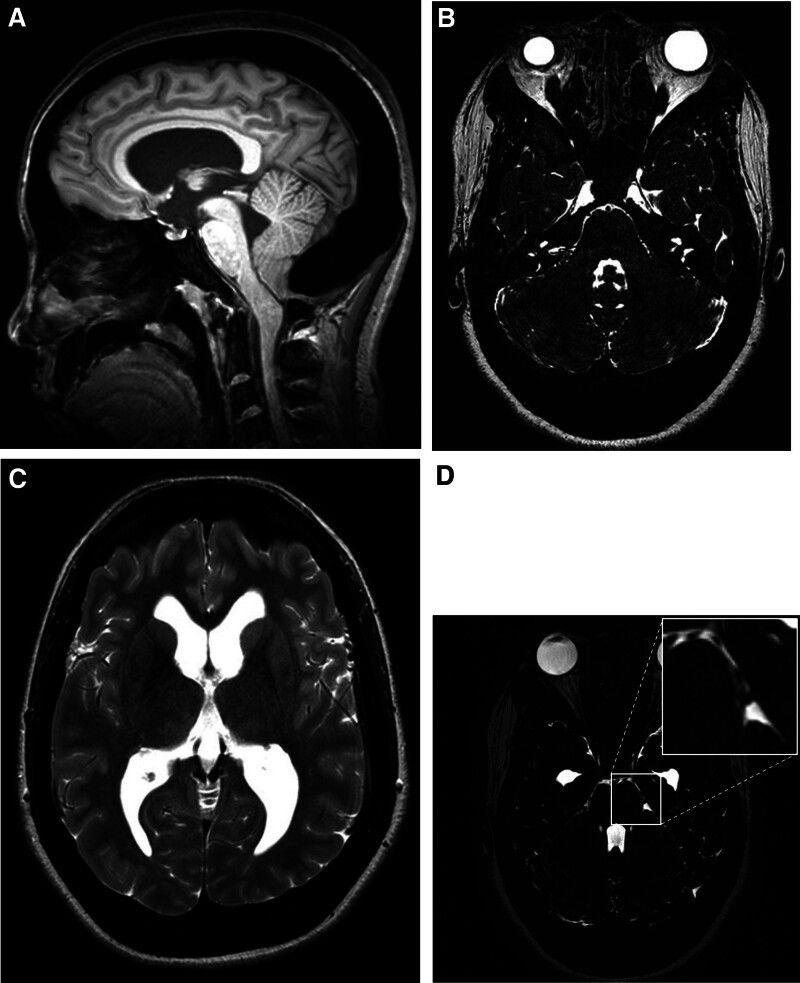
Magnetic resonance imaging prior to the first-stage decompressive surgery. (A) Sagittal T1-weighted image demonstrates posterior fossa crowding and brainstem compression caused by occipital bone overgrowth. (B) High-resolution T2-weighted image shows effacement of the cerebellopontine cisterns. (C) Axial T2-weighted image reveals mild ventricular enlargement, suggestive of obstructive hydrocephalus. (D) High-resolution T2-weighted image demonstrates limited visualization of the left trigeminal nerve and surrounding vessels due to severe cisternal crowding. The magnified inset highlights poorly defined neurovascular structures, raising suspicion for vascular compression of the trigeminal nerve.

Computed tomography (CT) performed before the first-stage surgery revealed diffuse cranial bone thickening with the characteristic “marble bone” appearance. The medullary cavities were obliterated, and calvarial thickness measured approximately 7 to 8 mm in the frontal region and up to 29 mm in the suboccipital area (Fig. 1). Magnetic resonance imaging demonstrated marked posterior fossa crowding due to occipital bone overgrowth and stenosis of the foramen magnum (Fig. 2A). This resulted in compression of the cerebellum and brainstem, with effacement of the cerebellopontine cisterns (Fig. 2B). Mild ventricular enlargement was also observed, suggesting obstructive hydrocephalus secondary to impaired CSF outflow (Fig. 2C). High-resolution T2-weighted sequences revealed an aberrant arterial loop near the left trigeminal nerve at the REZ, raising suspicion of vascular compression (Fig. 2D).

### 2.3. Treatment planning and surgical rationale

Because of the patient’s refractory TN and imaging findings demonstrating neurovascular compression, MVD was considered. However, severe posterior fossa crowding and foramen magnum stenosis limited surgical access and increased the risk of brainstem or cerebellar injury. Radiologic evidence of obstructive hydrocephalus raised concerns regarding potential neurological compromise. Although imaging suggested obstructive hydrocephalus, the patient exhibited no clinical features indicative of raised intracranial pressure, such as headache, nausea, vomiting, or papilledema. Therefore, direct intracranial pressure monitoring was deemed necessary.

A staged surgical strategy was planned to address these risks. The first-stage surgery aimed to decompress the posterior fossa and restore CSF dynamics, thereby facilitating safer access to the trigeminal nerve. Moreover, posterior fossa decompression might also reduce trigeminal nerve distortion and partially alleviate symptoms. After the intracranial space was restored and the surgical corridor widened, a second-stage MVD was scheduled under optimized anatomical conditions to achieve definitive neurovascular decompression.

Alternative treatment options, including continued medical therapy, CSF diversion (e.g., ventriculoperitoneal shunt), or ablative procedures such as radiofrequency rhizotomy, were considered but ultimately deemed suboptimal. The progressive nature of symptoms despite maximal medical therapy, combined with imaging-confirmed vascular conflict, warranted definitive surgical intervention. A single-stage MVD was not feasible owing to severe posterior fossa crowding and the risk of brainstem injury; thus, a staged approach was selected to optimize surgical safety and long-term outcomes.

### 2.4. First-stage surgery: posterior fossa decompression

**Figure 3.**
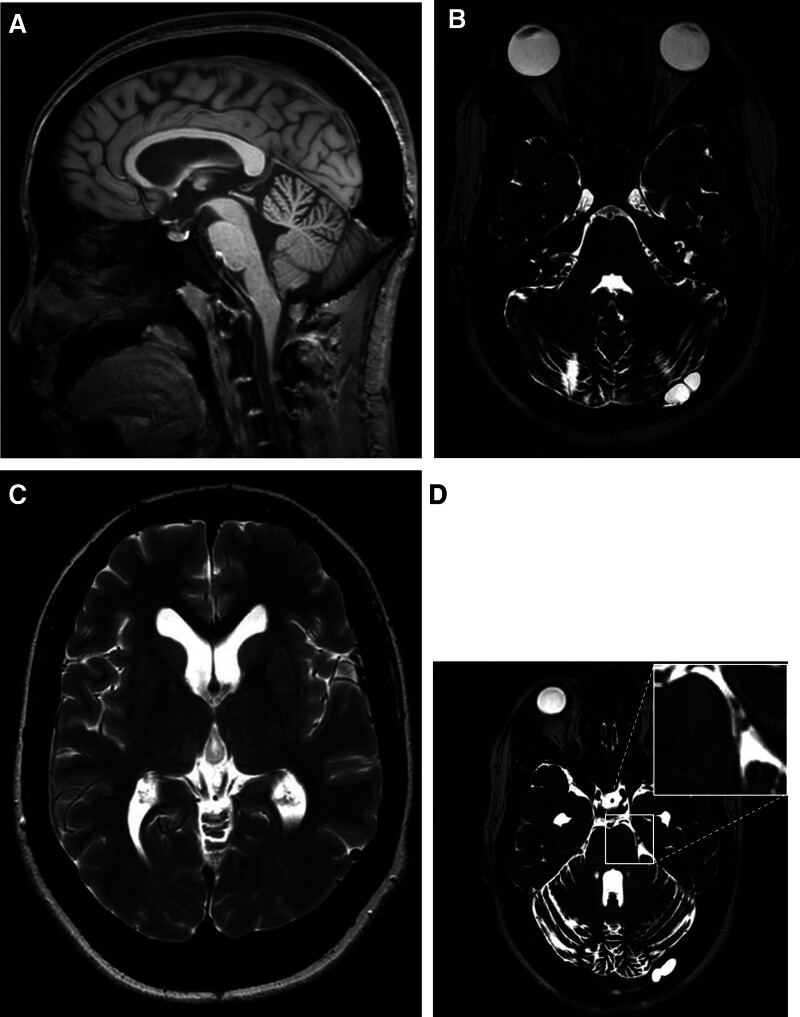
Magnetic resonance imaging following the first-stage decompressive surgery. (A) Sagittal T1-weighted image shows improved brainstem contour and posterior fossa expansion. (B) High-resolution T2-weighted image reveals restoration of the cerebellopontine cisterns. (C) Axial T2-weighted image shows reduced ventricular size, consistent with resolution of hydrocephalus. (D) High-resolution T2-weighted image demonstrates persistent neurovascular compression. The magnified inset highlights the neurovascular conflict at the root entry zone of the trigeminal nerve.

The first-stage surgery involved suboccipital craniectomy with C1 laminectomy to decompress the posterior fossa. Intraoperatively, the posterior fossa was markedly narrowed because of the thickened occipital bone, leaving limited space for cerebellar relaxation. Postoperative magnetic resonance imaging demonstrated restoration of CSF flow, re-expansion of the cerebellopontine cisterns, and decreased ventricular size, consistent with resolution of obstructive hydrocephalus (Fig. 3A–C). The patient reported transient improvement in facial pain; however, the symptoms recurred within 5 months, and follow-up imaging confirmed persistent neurovascular compression of the trigeminal nerve (Fig. 3D).

Histopathological examination of the thickened occipital bone showed diffuse cortical thickening with marrow obliteration and reduced osteoclasts, consistent with osteopetrosis. Given the transient postoperative symptom relief, clinical observation was initially pursued rather than immediate second-stage surgery. Upon recurrence of pain 5 months later, MVD was performed based on persistent symptoms and confirmatory imaging.

### 2.5. Second-stage surgery: MVD

**Figure 4.**
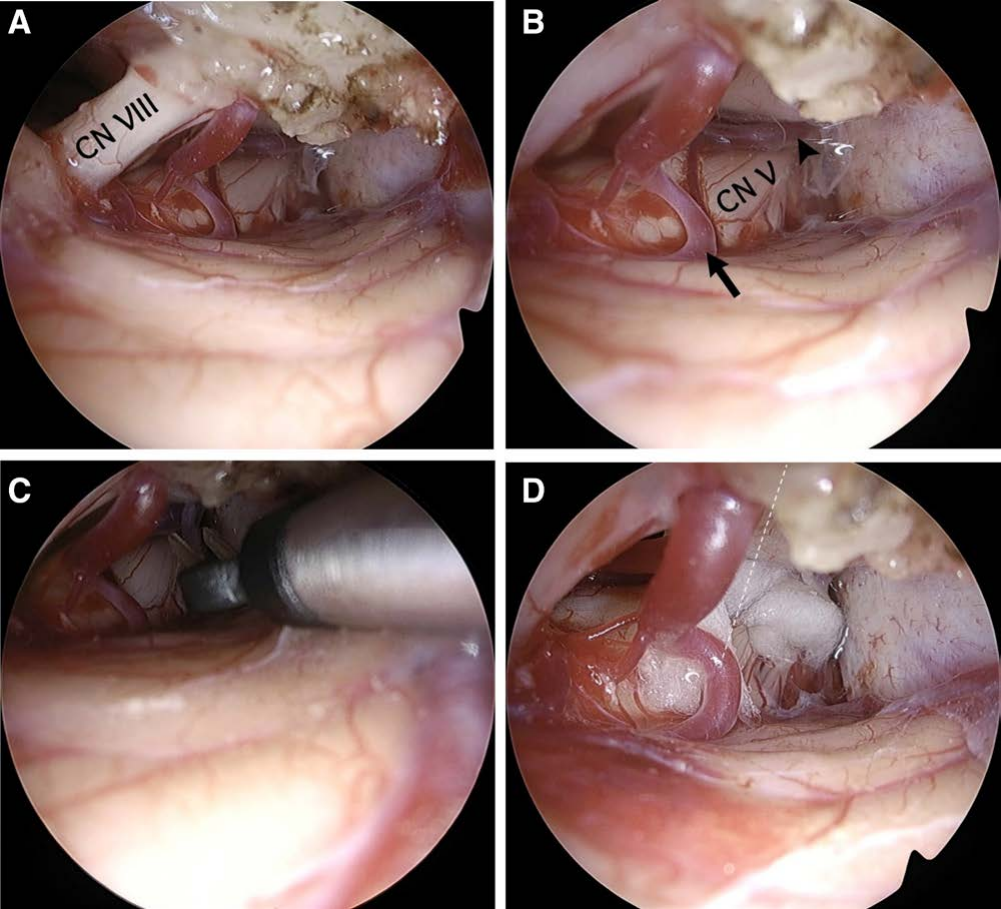
Intraoperative endoscopic views during the second-stage microvascular decompression(MVD). (A) Initial endoscopic view showing the expanded cerebellopontine angle after prior decompression, allowing safe and direct visualization of the trigeminal nerve. (B) Trigeminal nerve compressed by an offending artery (arrow) and an overlying vein (arrowhead). (C) Coagulation and division of the vein using bipolar cautery. (D) A Teflon pledget interposed between the artery and the trigeminal nerve, confirming adequate decompression.

The second-stage surgery was performed via a left retrosigmoid approach. The decompressed posterior fossa provided adequate space for gentle cerebellar retraction and improved visualization of the trigeminal nerve (Fig. 4A). Intraoperative brainstem auditory evoked potential monitoring was employed to preserve auditory function and enhance surgical safety.

Intraoperatively, an arterial loop, likely the superior cerebellar artery, was found compressing the trigeminal nerve at the REZ, accompanied by a large overlying vein (Fig. 4B). The vein was coagulated and divided using bipolar cautery (Fig. 4C), and a Teflon pledget was inserted to separate the artery from the nerve (Fig. 4D). Adequate decompression was confirmed, and the procedure was completed without complications.

### 2.6. Outcome

**Figure 5.**
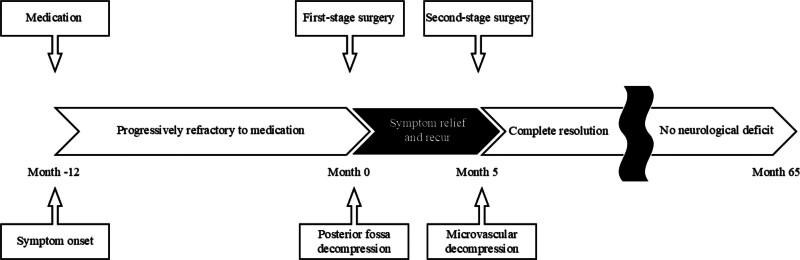
Timeline summarizing key clinical events and management milestones. A schematic overview of the patient’s clinical progression, including symptom onset, diagnostic workup, staged surgical interventions, and long-term follow-up outcomes.

Following the second-stage MVD, the patient experienced complete resolution of TN without neurological deficits or perioperative complications. Throughout more than 5 years of follow-up, she remained entirely pain-free, without recurrence, and had discontinued all medications. Notably, there was no facial numbness, paresthesia, or other sensory disturbance, and neurological examinations throughout follow-up consistently demonstrated intact trigeminal function. Serial imaging confirmed sustained decompression of both the posterior fossa and the trigeminal nerve, with no new abnormalities. A timeline summarizing the patient’s clinical course, surgical interventions, and long-term outcomes is illustrated in Figure 5. This long-term observation supports the durable efficacy and safety of the staged approach, as depicted in Figure 5.

## 3. Discussion

TN is a neuropathic pain disorder most commonly caused by vascular compression of the trigeminal nerve at the REZ.^[1]^ In typical cases, particularly among older patients, MVD yields excellent long-term outcomes when neurovascular conflict is identified on imaging.^[2]^ In contrast, TN in younger individuals warrants thorough evaluation for secondary causes, including tumors, demyelinating disorders, and craniofacial skeletal anomalies, such as osteopetrosis.^[3,4]^

ADO is a rare hereditary skeletal disorder caused by defective osteoclastic bone resorption, leading to diffuse sclerosis of the axial and cranial skeleton.^[5]^ Involvement of the skull base may result in cranial nerve compression through foraminal narrowing and distortion of adjacent neurovascular structures. Reported neurological complications include optic atrophy, hearing loss, facial palsy, and, rarely, TN.^[6,7]^ In ADO type I, neurological symptoms may develop insidiously despite minimal systemic manifestations.^[8,9]^ Bollerslev et al emphasized the importance of early recognition of cranial hyperostosis, as compressive neuropathy is often underdiagnosed in patients without overt systemic disease.^[8,9]^ In this case, the diagnosis of ADO type I was established based on characteristic radiologic and craniofacial findings, absence of hematologic abnormalities, and a clear autosomal dominant inheritance pattern. Although genetic testing was not performed, the clinical presentation was fully consistent with previously described diagnostic criteria for ADO type I.^[8,9]^

As summarized in Table 1, most previously reported cases of TN associated with ADO were managed conservatively or with ablative procedures such as radiofrequency rhizotomy. Although these approaches provide temporary pain relief, they frequently result in incomplete symptom control or permanent sensory deficits.^[1,10]^ Only a few reports have described successful MVD in this setting, largely because of the technical difficulties imposed by skull-base sclerosis and posterior fossa crowding.^[11]^

**Table 1:** Summary of previously reported cases of trigeminal neuralgia associated with autosomal dominant osteopetrosis, including treatment modality and clinical outcomes.

Report (year)	Patient details	Clinical features	Treatment approach	Outcome
Bollerslev et al, 1987	61/M, ADO	Craniofacial sclerosis TN (V3)	Conservative (medical)	No TN
Chindia et al, 1991	37/F, ADO	Craniofacial skeletal thickening TN (V2/V3)	Conservative (medical)	Partial relief
Kim et al, 1998	30/F, ADO	Foramen ovale and optic canal narrowing TN (V1-V3)	Percutaneous RF rhizotomy	Pain relief Facial numbness
Present case 2025	26/F, ADO	Craniofacial skeletal thickening, Posterior fossa crowding TN (V2, V3)	Staged decompression and MVD	Complete relief 5-yr follow-up) No deficits

ADO = autosomal dominant osteopetrosis, MVD = microvascular decompression, RF = radiofrequency, TN = trigeminal neuralgia.

This case illustrates a novel application of a staged surgical strategy, posterior fossa decompression followed by definitive MVD, in a patient with symptomatic TN secondary to ADO type I. Preoperative imaging before the first-stage surgery revealed marked occipital bone thickening, foramen magnum stenosis, obstructive hydrocephalus, and effacement of the cerebellopontine cisterns, resembling features of Chiari malformation.^[5,11]^

Posterior fossa decompression was initially performed to relieve brainstem crowding and restore CSF flow, thereby creating sufficient operative space for safe trigeminal nerve exploration. After optimizing intracranial dynamics and anatomy, the second-stage MVD enabled definitive neurovascular decompression under direct microscopic visualization.

This clinical sequence suggests that both cranial hyperostosis and vascular contact contributed synergistically to the patient’s pain. The transient improvement observed after posterior fossa decompression before MVD supports the hypothesis that mechanical compression and disturbed CSF flow significantly contributed to symptom generation. Accordingly, the staged approach was designed to address both mechanisms: first, by decompressing the posterior fossa to mitigate bony and CSF-related constraints, and, subsequently, by performing MVD to relieve vascular compression. Unlike previously reported cases that avoided or attempted MVD under constrained anatomical conditions, our 2-stage strategy proactively optimized the surgical environment, achieving a safe and durable outcome. Compared with prior reports that primarily employed conservative or ablative treatments for TN associated with osteopetrosis, this case demonstrates that, with meticulous preoperative planning and staged intervention, definitive MVD can be both feasible and effective in carefully selected patients with complex skull-base anatomy.

To our knowledge, no previous report has demonstrated complete and durable resolution of TN through a deliberately staged sequence of posterior fossa decompression followed by MVD in the setting of ADO. This case, therefore, provides new insight into surgical planning and supports expanding the role of MVD even in anatomically challenging conditions previously considered inoperable. In anatomically constrained patients with ADO, decompressive preconditioning can broaden the indications for MVD while minimizing the risk of neural injury. Our findings underscore the importance of detailed preoperative imaging, multidisciplinary planning, and individualized surgical sequencing in complex cases of secondary TN.^[3,6,12]^ Based on our experience, we propose that in patients with TN secondary to cranial hyperostosis, particularly those with posterior fossa crowding, a staged approach beginning with decompression to relieve anatomic constraints may enhance both the safety and effectiveness of subsequent MVD. Key decision factors include the degree of brainstem compression, CSF flow obstruction, and the presence of neurovascular conflict on imaging. Although additional cases are required to validate this strategy, our experience suggests that individualized surgical sequencing may broaden therapeutic options in complex TN associated with skull-base pathology.

This staged approach may also benefit from recent advances in microsurgical visualization. Techniques such as endoscope-assisted or exoscope-guided MVD have been developed to enhance operative exposure and reduce retraction-related injury,^[13]^ particularly in patients with limited posterior fossa space. Although endoscopic assistance was not feasible in our case because of severe anatomical crowding, such adjuncts may be valuable in appropriately selected patients. Furthermore, although large-scale studies of MVD in osteopetrosis are lacking, favorable outcomes have been reported in patients with Chiari malformation undergoing similar staged decompression followed by MVD.^[14]^ These observations conceptually support the feasibility and safety of this approach in patients with complex skull-base anatomy.

## 4. Limitations

As this is a single case report, the findings should be interpreted cautiously, and their generalizability to broader patient populations remains. Although this case demonstrates favorable long-term outcomes following a staged surgical strategy, its applicability is inherently constrained by the limitations of a single-patient observation. The decision to perform posterior fossa decompression followed by MVD was individualized based on this patient’s specific anatomical and clinical features. Therefore, extrapolation of this approach to other patients with ADO and TN should be approached judiciously. Selection bias is also possible, as patients with less severe posterior fossa constriction may not require a staged intervention. Future studies involving larger case series or multicenter data are warranted to validate the reproducibility, safety, and indications of staged decompression preceding MVD in comparable patients.

## 5. Conclusion

This case demonstrates that a staged surgical approach, posterior fossa decompression followed by MVD, can be a feasible and effective strategy for TN secondary to ADO. By sequentially addressing cranial hyperostosis and neurovascular compression, this approach achieved complete symptom resolution without complications, even in the presence of severe posterior fossa crowding. Careful patient selection, high-resolution preoperative imaging, and tailored surgical sequencing are essential for optimizing outcomes in anatomically complex cases. Clinically, this report suggests that a decompression-first strategy may provide a safe and pragmatic means of facilitating MVD in patients with TN and posterior fossa crowding caused by cranial dysplasia. This case reinforces the importance of individualized surgical sequencing and highlights the potential to expand MVD indications in patients previously considered inoperable due to skull-base anomalies. Beyond its surgical novelty, this case offers translational insight by proposing a structured, physiology-based decision framework that may inform future operative strategies in anatomically complex TN cases.

## Acknowledgments

We would like to thank Editage (www.editage.co.kr) for English language editing.

## Author contributions

**Conceptualization:** Hak Cheol Ko, Sungsoo Bae.

**Investigation:** Sungsoo Bae, Hee Sup Shin.

**Writing – original draft:** Sungsoo Bae, Seung Hoon Lim.

**Writing – review & editing:** Hak Cheol Ko, Hee Sup Shin.
